# Informing transplant candidate and donor education in living kidney donation: mapping educational needs through a rapid review

**DOI:** 10.1186/s12882-025-04116-0

**Published:** 2025-05-03

**Authors:** Tayler E. Truhan, James McMahon, Aisling E. Courtney, Paul Gill, Holly Mansell, Helen Noble, Joanne Reid, Nicola Rosaasen, Alison Wood, Clare McKeaveney

**Affiliations:** 1 School of Nursing and Midwifery, Queen’s University Belfast, 97 Lisburn Rd, Belfast, BT9 7BL UK; 2 Belfast City Hospital, Belfast Health and Social Care Trust, Belfast, UK; 3 University of Nottingham, Nottingham, UK; 4 College of Pharmacy and Nutrition, University of Saskatchewan, Saskatoon, SK Canada; 5 Queen Margaret University, Edinburgh, UK

**Keywords:** Living donor kidney transplantation, Rapid review, Qualitative evidence synthesis, Educational needs

## Abstract

**Objectives:**

Living donor kidney transplantation (LDKT) is a complex medical procedure requiring extensive education for both donors and transplant candidates. With technological advances in healthcare, video educational resources are becoming more widely used. This study aimed to synthesize the existing qualitative evidence on LDKT educational experiences, preferences, and needs from the perspectives of kidney transplant candidates and recipients, donors, and HCPs, to establish the essential LDKT education considerations for candidates and potential donors interested in kidney transplantation.

**Methods:**

A rapid review of qualitative studies on LDKT educational needs was conducted. A literature search was undertaken across MEDLINE, Embase, and CINAHL databases from 2013 to 2023. Cochrane Rapid Reviews Methods Group guidance was utilized.

**Results:**

Of 1,802 references, 27 qualitative studies were eligible for inclusion. Qualitative data was analyzed from 803 transplant candidates/recipients, 512 living donors, 104 healthcare providers, and 102 family/friends. Three main themes were identified, including Extensive LDKT Education Throughout Treatment; Shared Learning, Social Support, and Family Dynamics in LDKT; and Diversity and Inclusivity for Minorities.

**Conclusions:**

Improvements and innovations are needed regarding LDKT education for kidney transplant candidates, donors, and support networks.

## Background

A *Nature Reviews Nephrology* editorial published in 2024 highlights the rising global prevalence of kidney disease, surpassing all other chronic diseases currently prioritized by the World Health Organization [1]. Chronic Kidney Disease (CKD) is the final stage of kidney disease, in which the kidneys fail, and renal replacement therapy is needed. Renal replacement therapy options include kidney transplantation or dialysis. Dependent on the presence of clinical conditions and willingness of the patient, the best form of treatment for CKD is kidney transplantation due to enhanced patient outcomes and longer rates of survival [2, 3]. However, kidney transplantation is a complex medical procedure requiring extensive patient education. Patient education can aid patients and donors in making preoperative informed decisions, whilst improving medication adherence and self-management to maintain postoperative health [4–6]. A lack of knowledge regarding the procedure has been associated with a reduced willingness to donate [7], as well as postoperative complications including increased morbidity and mortality, and decreased quality of life [8, 9]. Living donor kidney transplantation (LDKT) is the most optimum for longer term outcomes, but this adds an additional layer of complexity, as it requires educating both transplant candidate and donor. Factors including economic deprivation, unemployment, and ethnicity, are independently and significantly reported to reduce the likelihood on an individual engaging in LDKT practices [10]. 

The most common form of education in kidney transplantation is usually a combination of one-to-one consultation with healthcare professionals (HCPs), group education sessions, and written educational materials such as leaflets or booklets [11]. These education sessions typically cover topics such as the benefits and risks of transplantation, pre- and post-transplant care, medication management, and lifestyle modifications. Some transplant candidates and donors may face difficulties with these forms of education, due to low general or health literacy and/or language barriers [12, 13]. Further, a critical review of LDKT patient information leaflets across thirty-nine UK renal units indicated patient information was ‘fairly difficult to read’, seldom included cultural and faith information, and scored on average 2.82 out of 10 for inclusion of information which supports shared decision-making [11]. In the US, data suggests that approximately 30% of patients may be uninformed about LDKT [14]. Additionally, donors in the US have reported feeling underprepared for the process of donation and potential post-donation complications [15]. In the Netherlands, some donors demonstrated a lack of knowledge about the risks of donation [16]. Beyond clinical information that is medically necessary to include, renal units may struggle to identify what additional information to incorporate in LDKT educational materials. This rapid review of qualitative studies on the LDKT educational needs and preferences of transplant candidates and donors will address this gap.

With technological advances in healthcare, educational videos are becoming more widely used, providing an effective way to educate candidates and donors about kidney transplantation, and improving accessibility through reducing travel requirements and associated costs for patients when compared to traditional face-to-face education and care [17, 18]. The inclusion of video content can act as a bridge to support health literacy by demonstrating complex medical information with animations [19] while also supporting alternate learning styles.

Video animation has proven a popular approach for the education of kidney transplant patients by colleagues in the USA [20–24]. Only two of these video animation series were comprised of LDTK components, and were limited to preliminary evaluations, thus lacking the robustness of a randomized controlled trial (RCT). However, these studies reported high levels of acceptability [20, 21, 24], as well as improvements in patients’ decisional self-efficacy, kidney allocation understanding [24], communication self-efficacy, and LTDK knowledge [22]. 

A prior RCT of video content on kidney transplantation for adults in Canada incorporated medical animations, patient testimonials, and HCP interviews [25]. Findings of this study found the videos improved transplant recipient knowledge and satisfaction and were regarded as an effective and practical approach to improving clinical education with minimal additional health care costs [26]. However, LDKT information was beyond the scope of the project. As LDKT is considered the optimal renal replacement therapy, content specific to this approach should form an essential component of pre-transplant education [27]. 

A 2017 quantitative scoping review identified evidence-based strategies to increase LDKT; [28] of these strategies, education directed at both the transplant candidate and their close social network proved to be most effective at increasing living donor evaluations and number of living donors. However, to our knowledge, there are no qualitative reviews which incorporate patient- and provider-identified LDKT educational needs. Extant qualitative research on the experiences of transplant candidates and recipients, support networks, and HCPs with LDKT can provide critical insights into how to improve LDKT education. Therefore, this rapid review supplements previous quantitative work by providing the first comprehensive summary of person-centered qualitative evidence on LDKT educational needs and experiences which can inform interventions and educational resources.

The aim of this rapid review is two-fold: (1) to provide the first synthesis of qualitative evidence on LDKT educational experiences, preferences, and needs from the perspectives of kidney transplant candidates and recipients, donors, and HCPs; and (2) to.

establish essential LDKT education considerations for candidates interested in kidney transplantation and potential donors, contributing to a vital gap in kidney transplantation education. A rapid review was conducted, rather than a systematic review, to inform LDKT educational materials currently in development. This review will provide a thematic synthesis of existing qualitative evidence on the educational needs of candidates and donors regarding LDKT.

## Materials and methods

A rapid review methodology was chosen as it allows for a time-sensitive, resource-efficient approach. This rapid review was conducted in accordance with Cochrane Rapid Reviews Methods Group guidance [29, 30]. Reporting of study identification is presented using the Preferred Reporting Items for Systematic Reviews and Meta-Analyses (PRISMA) 2020 diagram [31], as rapid review guidance is still in development [32]. 

### Eligibility criteria

Eligible studies were included if they met the following criteria: (1) Qualitative assessment of LDKT educational needs and/or experiences of adult kidney transplant candidates (pre-transplant), recipients (post-transplant), and/or living donors; (2) Participants over age 18 years; (3) Based in a high-income country, identified using The World Bank classifications [33], as health services across these countries are most comparable; (4) kidney transplant candidates or recipients, live donors, caregivers, or renal HCPs; (5) Full text available in English; (6) Published between 2013 and 2023. We limited the search to the last 10 years to focus on the most contemporary and innovative LDKT educational approaches and practices, as well as to maximise efficiency of the rapid review. Grey literature and review articles were excluded from the scope of this review.

### Information sources

Literature searches were conducted on October 24, 2023 using MEDLINE, Embase, and CINAHL databases. The search strategy was generated through consultation with a subject librarian (see Table 1 for example of MEDLINE search). Limits were applied for English language and the date range 2013-present. Reference lists of relevant existing reviews on LDKT were also mined.

**Table 1:** Search strategy for MEDLINE

*MEDLINE*	
1. ((kidney* or nephro* or renal) and (live or living) and (transplant* or donor or donation) and (educat*)).af. 2. limit 1 to (english language and humans) 3. limit 2 to yr=”2013–2023”	623 results

### Selection of sources

All citations were imported into Covidence systematic review software (www.covidence.org). Duplicates were removed by the software (*N* = 445). Source selection was performed by TT, with title and abstract screening followed by full-text screening. A streamlined screening process was followed according to Cochrane Rapid Reviews Methods Group guidance. The screening process was piloted among TT and CM prior to undertaking screening in its entirety, with a random sample of sources (*N* = 25). TT and CM then screened 20% of titles and abstracts (93.5% agreement) and resolved any conflicts. The remaining titles and abstracts were screened by TT, and 1,226 records were excluded at this stage. For the full-text screening stage, TT screened 138 full texts for inclusion. Included texts (*N* = 27) were confirmed by CM.

### Quality of reporting assessment

The Consolidated Criteria for Reporting Qualitative Health Research (COREQ) checklist [34] was used to assess the explicitness of reporting of included studies. The COREQ checklist was specifically created for qualitative research using interviews and focus groups. TT and CM assessed 25% of included studies using the COREQ framework and discussed any disagreements. TT then completed the assessment of the remaining studies.

### Data extraction

A data extraction form was utilized from the Cochrane Collaboration Qualitative Methods Group [35]. Data extraction was completed by TT and another researcher. CM verified the accuracy of extracted data. Data items relevant to the current review included: author, year, country, study aim, method, sample, context, approach to data analysis and interpretation, and qualitative themes.

### Synthesis of results

This rapid review utilized thematic synthesis, which involves a three-stage process to integrate multiple qualitative studies: (1) coding (TT), (2) construction of descriptive themes (TT and CM), and (3) development of analytical themes (TT and CM; confirmed by all authors) [36]. A thematic synthesis approach provides a deeper understanding of the educational experiences, preferences, and needs of transplant candidates and living donors [36]. Once the qualitative findings were extracted from each study, data was labeled through line-by-line coding. Coding consisted of pooling all the themes, representative text, and quotes identified in the included qualitative studies. No coding software was used; coding was completed using text highlights and labels/comments. In stage 1, codes were based on both pre-existing themes from the qualitative studies included in the review and new or revised concepts identified through the themes, quotes, and representative text. In stage 2, preliminary descriptive themes were developed deductively based on key results from the qualitative studies as well as inductively from patterns in the data. Similar codes were grouped together into preliminary descriptive themes around LDKT educational needs, some of which formed a hierarchical structure with subthemes. Ten descriptive themes were generated. Finally, stage 3 (thematic synthesis) was primarily inductive, as two authors (TT and CM) used descriptive themes to infer barriers and facilitators to LDKT education across populations in the included studies. This process formed the analytical themes and subthemes, by synthesizing the findings from the original studies [36], particularly in relation to our review aim to identify overall LDKT educational needs of both candidates and donors. The authors reviewed analytical themes in relation to descriptive themes. Analytical themes are visualized as a thematic map of LDKT educational needs.

## Results

### Literature search and study characteristics

**Fig. 1:**
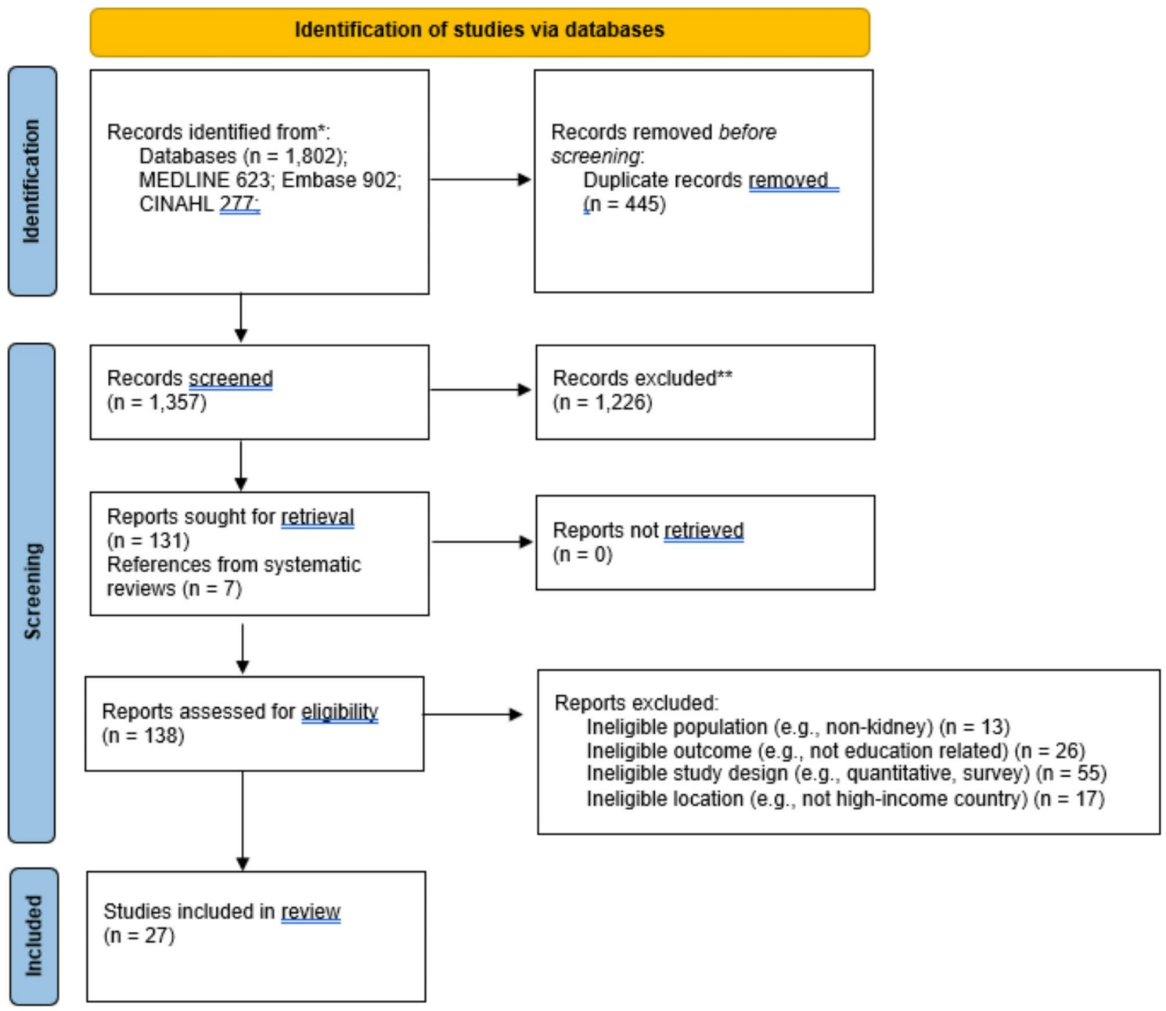
PRISMA flow diagram for study identification

Of the 1,802 references returned from our search, 27 were eligible for inclusion (Fig. 1). Qualitative data were analyzed from 803 kidney transplant candidates or recipients, 512 living donors, 104 HCPs, and 102 family/friends. All studies were concerned with LDKT education-related topics (e.g., decisional needs, barriers, solutions). Data were collected using semi-structured interviews, focus groups, workshops, and prompt-guided ‘storytelling’ (one study). Four studies included additional survey data. Studies were conducted across seven countries, including the United Kingdom, USA, Australia, Canada, the Netherlands, Sweden, and New Zealand. Study characteristics are presented in Table 2.

**Table 2:** Characteristics of studies included in the review (in alphabetical order)

Study	Country	Sample	*n*	Gender	Race/ Ethnicity	Data Collection	Analysis	Research Question/Aim	Findings/Themes
Ahmed et al., 2023	United Kingdom	HCPs: Transplant coordinators	19 8	11 F 8 M	73% White; 26% S. Asian	Semi-structured interview	Thematic analysis	Understand LDKT decisional needs of people from minority ethnic groups from perspective of kidney HCPs	Language Barriers; Cultural Awareness, Trusted Personnel, & Staff Diversity; Timing of Education; Setting of Education; Suitability of Patient Facing Information; Knowledge about LDKT; Risk Perception; Cultural & Religious Beliefs
		Nephrologist	7						
		Transplant surgeon	3						
		Specialist Nurse	1						
Alhalel et al., 2019	USA	Transplant candidates	21	18 F 18 M	86% Hispanic	Semi-structured interview; survey	Thematic analysis	Assess candidates and potential LDs perceptions of the Hispanic Kidney Transplant Program	Presented candidates and potential LDs perceptions and motivations, and impacts of Hispanic Kidney Transplant Program on LDKT
		Family members/ potential LDs	15						
Bailey et al., 2021	United Kingdom	Patients	13	17 F 15 M	84% White	Semi-structured interview	Thematic analysis	Views of stakeholders on possible intervention components using previously established LDKT interventions/support	Perceived Cultural Norms; Influence of Family on Decision Making; Resource Limitation; Evidence of Effectiveness
		Family members	4						
		HCPs	15						
Devitt et al., 2017	Australia	Patients	146	76 F 70 M	100% Indigenous	Semi-structured interview	Thematic analysis	Explore Indigenous Australian ESKD patients’ knowledge of, and attitudes towards, kidney transplantation	Interest in Transplant as a Treatment Option; Becoming Informed and Communicating with Clinicians and Carers; Family Support in Transplant Decision-Making; Negotiating Cultural Sensitivities
Getchell et al., 2017	Canada	Patients	13	10 F 9 M	*miss.*	Focus group	Thematic analysis	Provide insights from donors and recipients into the barriers faced during the transplantation process	Lack of Education for Patients and Families; Lack of Public Awareness on LDKT; Financial Cost to Donors; Health Care System-Level Barriers
		LDs	6						
Gordon et al., 2019	USA	LDs	17	15 F 3 M	100% African American	Focus groups	Thematic analysis	Assess African American donors’ perceptions of APOL1 testing	Decision-Making about APOL1 Testing; Information Needs about APOL1; Racial/Ethnic Identity; Insurance Coverage of APOL1 Testing
Hart et al., 2019	USA	Transplant candidates	28	12 F 16 M	50% White; 43.2% Black	Semi-structured interviews, focus groups	Thematic analysis	Describe what kidney transplant candidates understand about outcomes on the waiting list and the relative risks and benefits of kidney transplant options	Knowledge Gaps and Misperceptions; Desire for Frank, Individualized Information; Emotional Barriers; Decisional Support Needs
Ho et al., 2022	USA	Recipients	36	76 F 42 M	67% White; 8.5% Black	Prompt-guided stories	Thematic analysis	Identify common experiences and emotional changes shared by LDs and kidney recipients	Recipient: Struggling with Dialysis; Talking to Potential Donor; Feeling Guilty Accepting Donation; Feeling Happy when Someone Offered; Concerned for Donor’s Health; Being Able to Live Normal Lifestyle; Freedom from Dialysis; Closer with Donor; Recovery Process; Grateful for Donor Generosity LDs: Influenced by Religion; Influenced by Knowledge; Donated to Save a Loved One; Donor Family Concern; Fears and Challenges During Evaluation; Donors Would Donate Again; Happiness for Changing Patient’s Life; Becoming more Altruistic; Taking Better Care of Health; Recovery Experiences; Seeing Recipient Health Improve
		Donors	82						
Ismail et al., 2013	The Netherlands	Patients	50	25 F 25 M	20% Turkish; 20% Surinamese; 20% Dutch; 14% Moroccan; 14% Caribbean; 12% Cape Verdean	Focus groups	Grounded theory	Investigate the psychosocial and cultural factors that may constitute hurdles to LDKT	Patient Education: Lack of Tailoring, Poor Understanding; Cognitions & Emotions: Concerns & Misconceptions, Fears & Anxiety; Social Influences: Restrictions for Potential Donors; Communication: Patients’ Disinclined Attitude, Socio-cultural Factors
Kayler et al., 2020	USA	Potential LDs	32;	69 F 47 M	61%, White; 35% African American; 4% Other	Focus groups and individual interviews	Iterative process	Develop educational health animations on LKD	Suitability; Acceptability; Usability; Dissemination; Adaptability
		Friends/ family	10						
		Candidates & recipients	74						
Keddis et al., 2019	USA	ESRD/post Kidney transplant	12	5 F 7 M	100% Native American; 50% Navajo tribe	Semi-structured interviews	Thematic analysis	Investigate Native American patients’ perceptions and attitudes towards kidney transplants	Experience with kidney transplant education by the healthcare team; cultural beliefs regarding kidney transplant; personal motivation and attitude towards kidney transplant; financial burden of kidney transplant and post-transplant care; attitude about living donation.
Keller et al., 2022	USA	Patients	62	Patients: *Black* 13 M 16 F *Non-Black* 12 M 21 F Donors and carers: *Black* 3 M 3 F *Non-Black* 12 M 29 F	Patients: 47% Black, 53% Non-Black Donors and carers: 13% Black; 87% Non-Black	Descriptive study; secondary analysis of transcripts	Content analysis	Identify design and delivery adjustments for culturally adapting educational animations on LDKT originally developed for a diverse audience to better suit the needs of Black Americans	Cognitive barriers to LDKT: ambivalence; lack of knowledge; concern for donor Communication barriers to LDKT: reluctance to talk; difficulty talking KidneyTIME Videos as cognitive facilitators: attention-getting; efficient learning; manageable content; positive impact; new knowledge KidneyTIME Videos as communication facilitators: many dissemination channels; broadly shareable
		Donors	36						
		Caregivers	11						
King et al., 2020	USA	CKD (not yet on dialysis)	22; 6	13 F 9 M	100% African American	Semi structured in-person interviews	Thematic analysis	Understand African American patients’ knowledge of RRT options and how patient, provider and system-factors contribute to knowledge and preferences	Limited knowledge of home modalities and deceased donor options; CKD patients gave little thought to choosing RRT options; CKD patients relied on doctors for treatment decisions; while patients reported knowledge of LKDs it did not translate to receiving LKDT.
		ESRD and receiving treatment	6	5 F 1 M					
Lagging et al., 2022	Sweden	Potential LKDs	15	9 F 6 M	*miss.*; Swedish	Semi structured interviews.	Content analysis.	To investigate how people close to a person with kidney disease experience receiving a living donation letter.	Feelings evoked by the LD letter: the LD letter does not induce pressure to donate; the LD letter does not affect the relationship between the potential donor and patient; the LD letter makes the receiver feel like an important person. The LD letter creates clarification and trust: the LD letter clarifies that the decision to volunteer as donor is the letter receivers decision; the LD letter clarifies the patient’s phase in the transplant process; the LD letter unburdens the patient from approaching and informing potential donors. Opinions and suggestions about the LD-letter and further communication: a letter is preferred as the first step for communication regarding LKDT; opinions and suggestions regarding style and content of the LD letter; opinions and suggestions regarding follow up of the LD letter; needs for meetings about LKDT.
Manera et al., 2017	Australia/ Canada	LDs	123	45 F 78 M	74% White; 12% Asian/South Asian; 7% European	Focus groups	Grounded theory	Describe the expectations and experiences of LDs	Lacking Identification as a Patient; Empowerment for health; Safety net and reassurance; Neglect and inattention of care
Martin, 2014	New Zealand	Patients	193	87 F 104 M	52.9% New Zealand European; 23.5% Māori; 23.5% Pacific	Mixed methods; survey/ semi-structured interviews	Inductive analysis	Examine the preferences and concerns of New Zealand patients who are waiting for kidney transplantation	Preferences: prefer LKD to deceased donor transplant; willing to accept a LKD if offered; Concerns: donor health problems; painful donor recovery; loss of donor income; donor upset if transplant rejected
McKinney et al., 2021	USA	Candidates social support network	23	17 F 6 M	87% White; 13% Black	Focus groups	Inductive analysis	Characterize the barriers and facilitators social support network members experience in supporting transplant candidates	Advancing ESKD is a disease of the whole support network: Friends and families caring for patients with ESKD feel disconnected from other caregiver and stakeholder communities; families priorities first-hand knowledge about transplant options and expectations from recipients and their families. Unprepared to make decisions about transplant: difficulty communicating with their patients about ESKD and support roles; patients resisting help due to not wanting to feel like a burden; difficulty understanding the information; feeling helpless/needing guidance on how to help.
Pines et al., 2022	USA	LDs	25	16 F 9 M	Donors: 64% White; 24% Hispanic; 12% African American Recipients: 44.4% White; 33.3% Hispanic; 22.2% African American	Semi-structured interviews	Thematic analysis	Explore the decision-making and educational needs of KPD donors and recipients, compare the content being delivered to KPD recipients and donors, and discuss any KPD educational recommendations for improvement	Kidney paired donation awareness and decision making: becoming aware of paired donation; perceived benefits that motivated KPD participation; making the decision to purse KPD; quick decisions made with little information; systematic processing. Recommendations for improvement: key benefits of KPD addressing; key risks of KPD addressing; process to ameliorate challenges to KPD addressing; specifics of donor protection addressing; recommendations for educational improvement.
		Recipients	18	4 F 14 M					
Pronk et al., 2018	The Netherlands	Recipients	20	12 M 8 F	100% European	Semi-structured interviews	Inductive analysis	Investigate why patients with ESRD decided to solicit a living donor in public and what they experienced during public solicitation	Patients considerations preceding PS: Cautiousness in discussing living donation within social network; Reluctance to accept kidney from loved ones; Rejection/withdrawal of related donor candidates; Moral objections to paid donation; The ease of social media; Encouraged by others; Ends justifying the means; Despair and urge to take action; Public disclosure of vulnerability; Fear of being (perceived to be) selfish; Experiences with public solicitation: Positive emotions and support generated by action; Genuine and ulterior motives for donation; Patients acting as educators and screeners; Time- and energy-consuming process; Emotionally taxing process; Positive interactions with donor candidates; Feeling of dependency and obligation; Limited cooperation from health professionals; Demands a proactive attitude and media strategy
Ralph et al., 2019	Australia	Donors- recipient dyads (pre/post-transplant).	16	Donors 9 M 7 F Patients 11 M 5 F	81% Anglo-Celtic; 13% Other European; 6% Aboriginal	Longit., semi-structured interviews	Grounded theory/ thematic analysis	Collect longitudinal data on donor and recipient expectations and perspectives of their relationship in LDKT	Analytical decision making to mitigate regret; Donation as enacting familial responsibility for care; Strengthened interpersonal ties; Instability of relational impacts; Renegotiation social roles; Guilt over unmet expectations; Inevitability of the gift relationship
Ruck et al., 2018	USA	LDs	50	26 F 24 M	82% White	Semi-structured interviews	Thematic analysis	Explore and document qualitative insights into the apprehensions, misconceptions, and information-seeking behaviours of LDs in the US	Concerns about and experiences of donation among participants and their families; Taxonomy of donation concerns; Information participants desired prior to donating; Participants’ information-gathering behaviours
Schick-Makaroff et al., 2021	USA	Candidates	11	9 F 2 M	18% White; 9% African American; 9% Hispanic; 36% Asian	Semi-structured interviews; focus groups	Thematic analysis	Investigate the educational elements essential for facilitating an informed decision-making process among LDs	Education Is Contingent Upon and Motivated by Personal Circumstances; Education Is Supported Through Explanation of Risks and Benefits; Education Is Enhanced by Understanding the Overall Donation Experience; Education Is Personalized by Talking to Another Donor
		LDs	7	2 F 5 M	57% White; 29% Hispanic; 14% Asian				
Shaw, 2015	New Zealand	Directed LDs	19	13 F 6 M	*miss.;* New Zealand	Semi-structured interviews	Thematic analysis	Identify deficiencies in informed consent process concerning information disclosure and provide recommendations for improvements	Communication and psychological support; Information disclosure: timing of donation process; Post-surgery recovery and support
		Non-directed LDs	6	5 F 1 M					
Sieverdes et al., 2015	USA	Deceased Donor Recipients	16	14 F 13 M	100% African American	Focus groups/ surveys	Inductive/deductive	Explore perspectives of African American recipients regarding challenges, barriers, and educational needs in pursuing transplant	Concerns About Living Donors; Knowledge and Learning; Expectations of Support; Communication
		LD Recipients	11						
Skaczkowski et al., 2023	Australia	LDs	17	8 F 9 M	94% Australian; 6% Other; 6% Aboriginal or Torres Strait	Semi-structured interviews	Thematic analysis	Experiences of LDs residing outside metropolitan areas	Donor’s emotional well-being is influenced by the recipient’s outcome; varied levels of access to medical support and other important services in rural areas; travel takes a toll on time, finances and well-being; varied level of financial impact; medical, emotional and social challenges; both lay and health professional support is valued; varied levels of knowledge and experiences accessing information; a worthwhile experience overall
Traino et al., 2016	USA	LDs	81	55 F 55 M	67.9% White; 28.6% Black; 1.2% Asian/pacific islanders; 1.2% American Indian Alaskan; 1.2% Multiracial	Semi-structured interviews	Subgroup analyses/ Latent content analysis/ Statistical analyses	Assess donors’ perceptions of the information provided while considering living donation	Perceived Usefulness of Information; Desire for Additional Information; Understanding of and Satisfaction with Information; Subgroup Analyses for Ethnicity, Education, and Income
Waterman et al., 2020	USA	Patients	40	19 F 15 M 6 *miss.*	22.5% White; 50% Hispanic; 12.5% African American; 15% East Asian/Pacific Islander/ Other/	Structured interviews	Grounded theory	Examine barriers to transplant education and preferences based on CKD stage and primary language spoken	Perceived Barriers to Transplant: Confusion about CKD; Lack of transplant knowledge by family/friends; Lack of knowledge about appropriate LDs and fear of risk to LD; Fears about risks of transplant; Fears of being a burden; Financial burdens of ESRD and transplant; Transportation and scheduling challenges; Difficulty using interpretive and medical services for Spanish speakers; Hispanic patients’ medical mistrust and specific concerns about risks for LD; Primary responsibility to pursue transplant on the patient. Transplant Education Preferences and Recommendations: Earlier introduction to transplant education with prevention focus; Benefits of transplant and recovery; How to get on the transplant wait-list; Financial resources for transplant; LDKT information for patients and support persons; Transplant education geared toward family and friends; Offer in-person classes with online options; Make educational resources available in Spanish and classes taught by bilingual educators; Culturally sensitive education; Increase support for patients post-class
		Support network	13	7 F 4 M 2 *miss*.	31% White; 15% Hispanic; 8% African American; 46% East Asian/Pacific Islander/ Other				
		HCPs	10	*miss.*	*miss.*				

Note. Some studies did not report gender other than Female, so there may be participants who were non-binary or did not report gender. *miss.* = missing; HCP = healthcare provider

### Comprehensiveness of reporting

Of the 32 items included in the COREQ checklist, 100% of studies reported description of sample and data and findings consistent, and 96.3% of studies covered sampling methods, sample size, audio/visual recording, derivation of themes, and quotations (Table 3). No studies reported conducting repeat interviews.

**Table 3:** COREQ 32-item checklist of included studies

COREQ item	Studies reporting item	*n* (%)
1. Interviewer/facilitator	[38, 40, 42, 44, 48, 50, 57, 59–63]	22 (81.5%)
2. Researcher credentials	[28–33, 35–63]	24 (88.9%)
3. Researcher occupation	[29, 31–33, 35–63]	21 (77.8%)
4. Researcher gender	[42, 47, 50–54, 56, 59, 61–63]	6 (22.2%)
5. Researcher experience	[29, 31–33, 35, 37, 38, 40–42, 44–48, 50–57, 59–63]	18 (66.7%)
6. Relationship established	[29, 33, 38, 42, 43, 47, 51–53, 56, 62]	6 (22.2%)
7. Participant knowledge of researcher	[42, 47, 51, 53, 54, 56, 62, 63]	4 (14.8%)
8. Interviewer characteristics	[29, 31, 33, 35, 36, 38, 40–42, 44–48, 50–57, 59–63]	17 (63%)
9. Methodological orientation	[29, 30, 33–35, 39, 42, 45–49, 51–63]	17 (63%)
10. Sampling	[28–63]	26 (96.3%)
11. Method of approach	[29–35, 37–63]	21 (77.8%)
12. Sample size	[28–42, 44–63]	26 (96.3%)
13. Non-participation	[30, 31, 33, 35, 40, 42–63]	14 (51.9%)
14. Setting of data collection	[29, 31–36, 38–43, 45–63]	22 (81.5%)
15. Presence of non-participants	[35, 41, 44, 47, 50, 52, 56, 61]	4 (14.8%)
16. Description of sample	[28–63]	27 (100%)
17. Interview guide	[29–63]	23 (85.2%)
18. Repeat interviews		0 (0%)
19. Audio/visual recording	[29–35, 37–63]	26 (96.3%)
20. Field notes	[36, 37, 44, 45, 53, 54, 58–60, 62]	8 (29.6%)
21. Duration	[30, 33, 35, 39, 42, 44–49, 51–63]	19 (70.4%)
22. Data saturation	[30, 33, 35, 39, 42, 44–63]	17 (63%)
23. Transcripts returned	[29, 38, 41, 50, 52, 61]	3 (11.1%)
24. Number of data coders	[29, 30, 32, 33, 35, 38–63]	22 (81.5%)
25. Description of coding tree	[30, 35, 38, 39, 45, 47, 51, 54, 58–60, 63]	9 (33.3%)
26. Derivation of themes	[28–39, 41–63]	26 (96.3%)
27. Software	[28–35, 37–44, 46–48, 52, 53, 55, 56, 58–62]	19 (70.4%)
28. Participant checking	[36, 45–47]	2 (7.4%)
29. Quotations presented	[28–63]	26 (96.3%)
30. Data and findings consistent	[28–63]	27 (100%)
31. Clarity of major themes	[29–63]	26 (96.3%)
32. Clarity of minor themes	[33, 35, 36, 38, 39, 42, 44, 45, 46–48, 50, 54, 55, 59, 63]	8 (29.6%)

### Synthesis

**Fig. 2:**
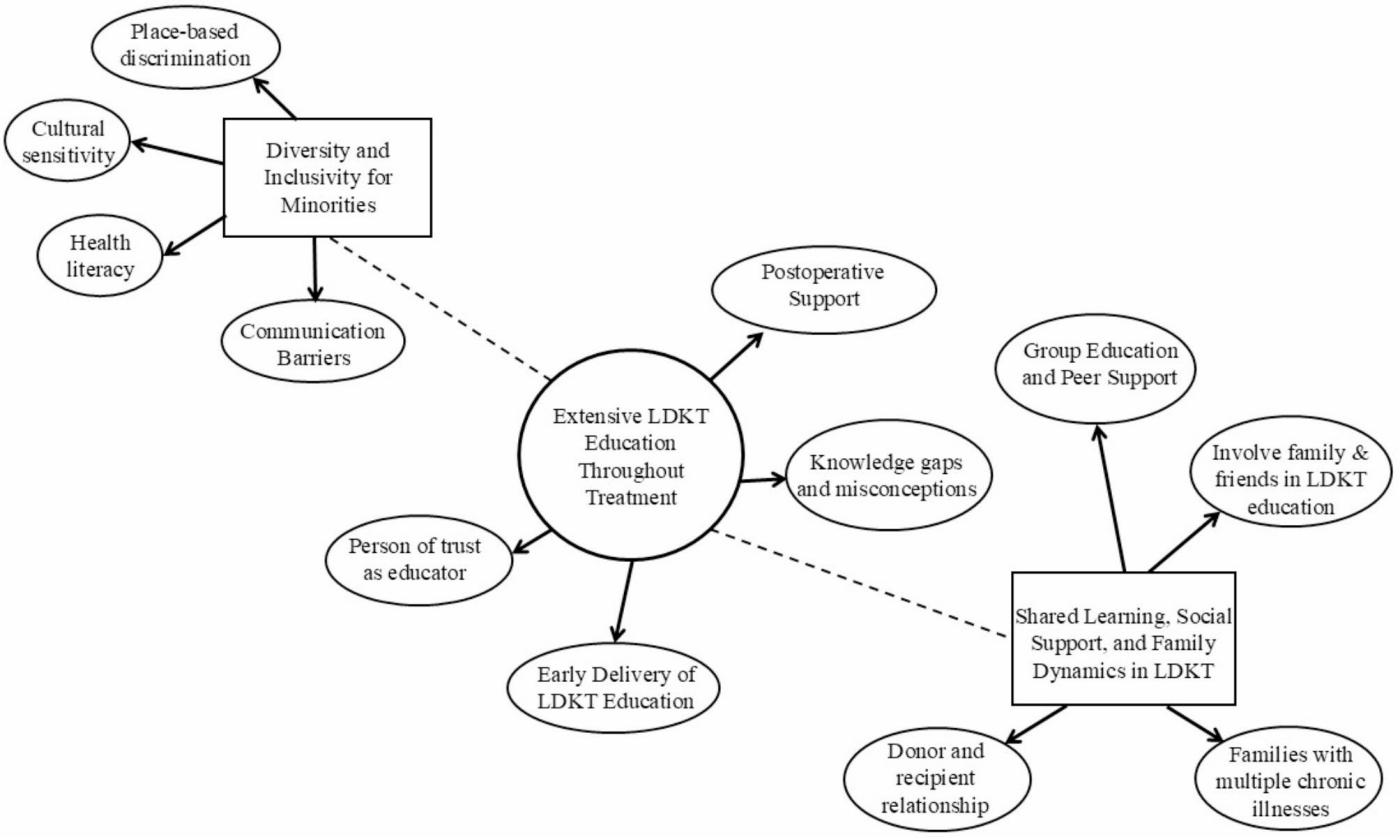
Thematic Map of LDKT educational needs of recipients and donors

From 27 studies, we identified three major themes including (1) Extensive LDKT Education Throughout Treatment; (2) Shared Learning, Social Support, and Family Dynamics in LDKT; and (3) Diversity and Inclusivity for Minorities. Illustrative quotations are presented in Table 4. A thematic map of LDKT educational needs is presented in Fig. 2. Theme 1 occurred in all studies included in this review, and as such, is linked to the other three smaller main themes (visualized as dotted lines in the figure) which occurred in varying numbers of studies. All three main themes comprised several subthemes, as indicated by arrows in the figure.

**Table 4:** Illustrative quotations of themes

Themes & subthemes (in italics)	Quotations	Contributing references
**Extensive LDKT education throughout treatment**	*I did have fears and questions about the process…What are the odds that the surgery will work and that my dad’s body will accept my kidney? (Donor)*	[37–63]
*Knowledge gaps and* *misconceptions*	*And I just felt so helpless and it’s been so many times where, as a family member, I feel helpless because I think, if I could go on there as a family member and go okay, here’s information. *[56] *I did want to know how that works…was everybody going to go on the table at the same time? Do they take out all the kidneys at once? How does that work? (Recipient) *[59]	
*Person of trust as* *educator*	*…I would love to get the information from a person that was getting dialysis and…from a renal doctor and my primary doctor. If I needed that type of information, I would contact that type of doctor first. (69-year‐old female, CKD Stage 3) *[47]	
*Early delivery of LDKT* *education*	*If someone offers, I’d certainly run with it, especially after being on dialysis for the time I’ve been on now…At the time I wouldn’t take one off that person because they’re a pain in the arse, but it’s got to the stage now where there was that discussion [in the media] about the paedophile or some guy that wanted to donate. Someone asked me, ‘‘Would you take it?’’ In a flash, yeah. They said, ‘‘Oh wouldn’t you feel…?’’ I said, ‘‘You haven’t been on dialysis for day after day, month after month, year after year. Because if you had, you wouldn’t think twice. *[52]	
*Postoperative Support*	*Maybe it’s not sort of mandatory, I know every doctor’s gonna have their own opinion on things*,* but it just would be nice to know how you’re meant to look after yourself afterwards. (Woman*,* Donor*,* 40s) *[58] *Get good support for yourself afterwards*,* cos there’ll be a lot of support for the recipient. Make sure you’ve got someone that will care for you. Because that’s important too. *[51]	
**Shared Learning**,** Social Support**,** and Family Dynamics in LDKT**	*Now my family are talking about a transplant. They need some information. We need to talk together about this and we all need information about what donating a kidney involves. It is a bit hard to talk about it though because my family doesn’t get together that often. *[38]	[37–50, 52–63]
*Donor and recipient* *relationship*	*As far as sex goes*,* I am frightened to have sex because I’m on the immunosuppressants*,* every time I have sex I get a urinary tract infection. And I just don’t want them there*,* they’re too horrible and so I always decline it. (Female; Spousal recipient) *[42]	
*Involve family & friends* *in LDKT education*	*I just tried to get fully educated on it*,* as did my family… It lessened all the concerns a lot to the point where there wasn’t a lot of concern going into it. *[53]	
*Families with multiple* *chronic illnesses*	*I mean especially when you’re talking years. Now not only is that one person affected*,* it’s affecting the whole family. And from you on down to your children or whatever. Everybody is affected*,* not just you and your spouse. It’s going to be everybody. (Female) *[59]	
*Group education*	*I didn’t really listen to other people*,* but when I saw that fella [who’d had a transplant]*,* I looked at him and said*,* ‘‘How long you had your kidney?’’ He said*,* ‘‘Eight years*,*’’ and he’s still going so that made me think again. *[37]	
**Diversity and Inclusivity for Minorities**	*Let’s put the effort in*,* before we approach patients*,* to get them to engage we need to know how living donation sits within their culture” (Female*,* Specialist nurse). *[40]	[38–40, 41, 44, 43,46,47, 51, 52, 54–56,58–61,63]
*Health literacy*	*That would have been helpful. You know*,* videos and courses and things like that*,* you know the coordinators*,* to whom you‘re connected. I felt like they just did not have the bandwidth to be responsive to questions. So*,* if I could have found the answers myself*,* then that would have been easier. (Recipient) *[52]	
*Cultural sensitivity*	*“things that most Hispanics do not know*,*”…“very important because*,* as Hispanics*,* we have many myths that are harmful… and they showed us that it is nothing like what people say.” *[54]	
*Place-based* *discrimination*	*I’m very lucky that we have a pathology department*,* or collection centre*,* in [nearby regional town]. For a population of only 1200 people*,* we’re truly gifted. Yeah*,* just go around the local medical centre when they want all these blood samples.*[51]	
*Communication barriers*	*They [staff] don’t give it [information] the right way. Instead of like trying to teach them*,* they come across like they know everything and they don’t compromise on that*,* hey? When they come across like that everyone’s too scared to ask them questions why*,* so then they just shut up and think*,* “Well I’ve been told this*,* so that must be it. *[38, 39]	

### Extensive LDKT education throughout treatment

A main theme was Extensive LDKT Education Throughout Treatment, with all studies highlighting a need for this in LDKT education. Four subthemes were identified, including knowledge gaps and misconceptions, person of trust as educator, early delivery of LDKT education and postoperative support.

A key concern was addressing the knowledge gaps or misconceptions of recipients and donors, particularly recipients’ skewed risk perceptions for donors [37, 38]. Some transplant candidates would not consider asking family to donate due to their perception of risk to the donor [39]. HCPs felt potential recipients had genuine concerns about how someone could live well with one kidney [40]. For caregivers, lack of knowledge contributed to feelings of helplessness, as they felt less able to identify practical ways to support recipients. Many candidates and donors wanted more information on the benefits of LDKT, such as longer graft survival [39], and transplant in general, including freedom from dialysis [41] and greater social participation [42, 43]. Recipients suggested they would have been open to LDKT sooner if they understood the benefits, and the realities of life on dialysis [37]. Donors also wanted to know the potential benefits to recipients of LDKT [44]. 

Knowledge gaps included timeline for evaluation [45], matching and eligibility of donors [46], wait-listing for transplant [43], changing treatment modalities (i.e., switching from dialysis to transplant) [47], what surgery and hospitalization for transplant was like [46], and recovery after transplant [48]. Donors also wanted to know about the recovery process [41, 46, 48–53]. Some recipients and donors experienced frustration due to lack of knowledge about the transplant process [38], and with long evaluation processes pre transplant or donation.

Recipients, donors, and caregivers also wanted more information on kidney-paired exchange and altruistic donation [46]. Recipients and donors who participated in kidney paired donation highlighted the experience of helping multiple people [52], and the flexibility for donors in terms of scheduling. However, some felt a lack of control about where their donated kidney was going. Donors and recipients wanted more information on the roles of different organizations involved in kidney donation and transplantation (e.g., the National Kidney Registry in the USA) [52]. 

Many donors and recipients wanted a ‘person of trust’ as the LDKT educator, such as a physician. Patients from minority ethnic groups indicated they trusted LDKT information given by a HCP more, as a physician has “*first-hand information*” and “*is not going to trick you*” [54]. Information delivered by HCPs can address incorrect assumptions about kidney donation and transplantation and reduce patient burden [55]. Although trusting of information provided by HCP’s, individuals from minority ethnic groups often possess a distrust towards the overall healthcare system, with community resources, such as churches or cultural centers, proving advantageous in breaking down such barriers [54, 56]. HCPs should deliver transplant education early on. Some candidates and recipients felt they did not have time to prepare for end-stage kidney disease and needing a transplant [43], and wished they had been informed earlier about the realities of dialysis [37]. Candidates and recipients suggested taking a prevention focus to transplant education, including support for candidates who need assistance in identifying a donor [43, 45]. HCPs also observed patients can become over-loaded with information in short spans of time, and LDKT options should be discussed early on [40]. 

A subtheme, postoperative support, emerged through the thematic synthesis. Donors, recipients, and caregivers were concerned with the impacts LDKT may have on other areas of life outside of physical health and wanted recommendations on how to achieve a healthier lifestyle long-term. Two subthemes were identified, including available support and resources and lifetime healthy behaviours. Recipients and donors were particularly concerned about the financial impacts donation might have [37, 40, 45], especially if donors lived in a rural area [51]. Other concerns were related to health insurance [49], how long recipients or donors would be out of work [43], or paying for external specialists postdonation [48]. Some patients expressed confidence in their healthcare access or income capacity [56]. Recipients and donors wanted to know about other available support, such as counsellors or social workers [52, 57], as well as the need for more follow-up appointments [48, 51]. 

Further, recipients wanted to know about anti-rejection medications [39], including possible reactions. Caregivers were also concerned about the impact of medications on the recipient’s mood [42]. Donors wanted to know about any issues they might have with pain medications and recommendations for diet and physical activity [48]. African American donors and recipients wanted further information on long-term impacts of Apolipoprotein L1 (APOL1) gene variants, which are associated with increased risk for kidney disease, and recommended lifestyle changes [58]. 

### Shared learning, social support, and family dynamics in LDKT

The theme ‘Shared Learning, Social Support, and Family Dynamics in LDKT’ encompassed issues or changes in the donor and recipient relationship, recipients and caregivers wanting families and/or friends included in LDKT education, considerations for families with multiple chronic illnesses, and the value of group education and peer interaction. This theme was discussed in 26 of the 27 studies.

Donors, recipients, and caregivers expressed an interest in speaking with their peers about LDKT [50, 53, 59], and recipients were interested in receiving group education either in the hospital or at home [39, 45, 54, 55, 60]. Speaking with peers who had received a transplant was the impetus for some candidates to consider LDKT. Several studies also discussed engaging with the public (e.g., public awareness campaigns) [45, 59], which might contribute towards greater societal and peer acceptance of candidates who use public solicitation for a kidney donation [61]. 

Many potential recipients were wary of feelings of tension, decisional regret, and guilt post donation, which made them reluctant to accept a kidney from their spouse or family [46]. Prospective donors who were caregivers wanted more education about the role of a caregiver beyond typical physical tasks, such as attending clinic and “being an extra set of ears and making sure you heard everything correctly” [46]. Donor-recipient pairs in the same household (e.g., spousal donors) who did not have additional support highlighted particular difficulties with the recovery period, as donors were in recovery and providing care for recipients [48]. Other unexpected difficulties related to physical intimacy, discrepant energy levels, being in a ‘gift’ relationship (i.e., recipient feeling indebted to donor), and unanticipated caregiving responsibilities post-donation due to post-operative complications [42]. The donor experience also helped strengthen relationships [41], and enabled some recipients and donors to do more activities together [42]. 

Recipients wanted HCPs to communicate with their support networks and provide additional resources for facilitating friends and family understanding their diagnosis [62], as some reported a lack of knowledge within their support networks [43]. Potential recipients stated educating their family quelled donation fears [41]. This was highlighted as important as certain family members might influence other family not to donate, thereby limiting a recipient’s potential donor pool (e.g., brothers’ spouse influenced him not to donate) [55]. Caregivers wanted to be involved in education so they could better support the person with kidney disease [59]. Some candidates had family members who had other chronic comorbidities (e.g., diabetes), and did not want to ask their adult children to donate [38]. There were also families who had a history of kidney disease, with multiple family members in need of a transplant [59].

### Diversity and inclusivity for minorities

Diversity and Inclusivity for Minorities was based on 16 of the 27 included studies. This theme included four subthemes, comprising a need for greater focus on health literacy and literacy in general, cultural sensitivity, place-based discrimination (i.e., rurality of some patients), and communication barriers that recipients and donors may face.

HCPs in the UK highlighted that much of the take-home information provided in leaflets may not be appropriate for candidates and donors with low health literacy [40]. Patients suggested using different formats, such as providing more video education resources, which can promote self-education [46, 52]. Candidates and recipients also expressed educational needs related to language barriers [40], difficulty communicating with HCPs (e.g., feeling intimidated) [38], and fear of rejection from potential donors [39, 60, 61, 63]. HCPs suggested hospital interpreters were best suited to supporting non-native speaking patients due to the complex nature of some medical terminology [40], whereas some patients were wary interpreters would not know as much as the physician and would convey incorrect information [54]. Candidates feared negative reactions from support networks about donation, and did not know how to begin the donation conversation [63]. Caregivers felt communicating with candidates was difficult when they withheld information related to their kidney disease [59]. 

Possible mistrust of the healthcare system [60], past mistreatment [40], stigma associated with issues such as being APOL1 positive [59], and taboos around speaking about illness or organ donation should also be considered [39]. Candidates may have certain cultural beliefs or myths about organ donation that make them wary of accepting a living donor [40]. Hispanic patients indicated it was important for education to be culturally competent and sensitive [54]. Recipients and donors from rural areas experienced greater difficulties accessing education and appointments. Donors missed follow-up appointments due to travel difficulties [48]. Rural patients had variable access to local resources [51]. Some patients suggested providing occasional home education would help in terms of costs associated with travel, as well as discomfort in clinical, unfamiliar environments [55]. 

## Discussion and conclusion

### Discussion

This rapid review aimed to synthesize the existing qualitative evidence on LDKT educational experiences, preferences, and needs from the perspectives of kidney transplant candidates and recipients, donors, and HCPs, to establish the essential LDKT education considerations for candidates and potential donors interested in kidney transplantation. A total of 27 qualitative studies, conducted between 2013 and 2023, on LDKT educational needs of candidates and donors, including diverse perspectives of HCPs, transplant candidates and recipients, living donors, and family members and friends were included in this review.

A key theme uncovered through the analysis, ‘Extensive LDKT Education Throughout Treatment’, occurred in all included studies. Findings indicate a desire for education to address post-operative concerns, including feelings of guilt post-donation, the potential impact of donation on life and relationships, and the role of the caregiver. As evidenced in studies included in the Extensive LDKT Education Throughout Treatment theme, if the process and timeline to kidney transplantation is made clear at the start of LDKT education and delivered by a person of trust, recipients or donors may be less likely to experience negative emotions or repercussions. This is associated with the need for earlier delivery of LDKT education in the kidney disease pathway. The 2014 American Society of Transplantation Consensus Conference covering best practices in LDKT listed early and consistent LDKT education as one of the highest priorities [64]. 

One of the main barriers to LDKT, and subsequent need for extensive education, is recipient misconceptions about the physical risks to donors. This may be true, particularly for ethnic minorities who could also experience mistrust of the healthcare system and HCPs [65]. In a study with ethnic minority transplant candidates in the Netherlands, candidates indicated they would not consider asking a family member for a kidney due to their perception of risk to the donor [39]. A 2013 qualitative synthesis review of studies on expectations and attitudes of candidates towards LDKT generated a theme around patient guilt and responsibility for potential kidney donors [66], supporting the need to address this in LDKT education. Misconceptions may also be tied to cultural norms or ideologies surrounding organ donation [67], highlighting the need to develop culturally sensitive and diverse education.

In addressing the misconceptions of risk, it is important to also stress the benefits of LDKT to both recipient and donor. Understanding the scope and experience of kidney disease, including the realities of dialysis [37], and the positive impact that transplantation can have regarding graft survival and physical health of the recipient [68], as well as increased social participation for donor-recipient couples and a decrease in caregiving responsibilities [42–44], may increase LDKT acceptability. LDKT may also improve health-related quality of life for both donor and recipient, as the majority of recipients and donors experience positive outcomes post-LDKT [69, 70]. 

Potential barriers towards the success of delivering education were identified within the ‘Diversity and Inclusivity for Minorities’ theme, including poor health literacy, communication barriers, place-based discrimination, and cultural considerations. It is essential that barriers towards the delivery of LDKT education are considered to ensure the success of educational interventions. Engaging with religious organizations has been cited as an appropriate approach for breaking down cultural barriers [71], and thus may be implemented during the development of education content. Further, previous LDKT interventions for black and/or Hispanic kidney patients have successfully delivered education and reported improvements in LDKT knowledge [72–74]. The delivery of educational content to meet the specific needs of culturally diverse populations can add a layer of complexity during the development of educational interventions, potentially requiring the need for individual interventions to be developed based on the population of interest.

The importance of improving LDKT education delivery in rural areas has long been recognized. Delivery of home-based education is one such way to remove barriers associated with place-based discrimination, whilst also benefiting patients from different socio-economic backgrounds and ethnic groups [75]. In addition, video technology has been recommended as a means of successfully delivering education to those suffering from place-based discrimination [7, 71], potentially supporting the approach of video animation that has proven successful previously.

Within the ‘Shared Learning, Social Support, and Family Dynamics in LDKT’ theme, group education sessions including candidates and recipients at all stages of CKD and their support networks should be offered earlier on in the kidney disease pathway. This will give candidates and potential donors time to understand the next steps involved in kidney disease treatment, including the possibility of LDKT. Transplant candidates and donors may also wish to take the time to do their own independent research [52, 60], and have indicated that hearing from peers who are further along in the kidney disease pathway is particularly beneficial [37]. Group education sessions might be supplemented with virtual peer-support resources such as The Living Donation Storytelling Project [76]. By increasing involvement of family and friends in LDKT education, and including them in conversations surrounding organ donation, social support networks will have greater understanding of the potential far-reaching benefits of LDKT beyond the recipient’s physical health.

The findings of this review support existing research which highlights the need for improved patient education regarding LDKT [11, 14, 77]. A 2015 review found relatively few studies which addressed best practice regarding CKD transplant education– defined as ‘clear, comprehensive, understandable, and motivating to facilitate patients successfully completing the clinical steps necessary to be evaluated for transplant’ [78]. A cornerstone of high-quality health and social care research, including patient education development, is patient and public involvement and engagement [79], which is now required by many funders of health and social care research globally. The themes and subthemes from this review may therefore serve as topics for stakeholder input regarding LDKT education development, and can inform more ‘clear, comprehensive, understandable, and motivating’ materials.

Regarding LDKT education development, multimedia resources for patient education and kidney disease care are on the rise [17, 18]. Resources which combine video, animation, and text may help to address communication barriers related to difficulty understanding complex medical topics, speaking with HCPs, and discussing organ donation with social support networks. They can be disseminated to transplant candidates and donors early in the kidney disease pathway. Candidates and donors may revisit educational topics they find complex, and videos can incorporate storytelling or testimonials from other recipients and donors. Animations, testimonials, and videos can achieve better representation of diverse patient populations and can provide translated text for non-native speakers [25, 63]. Therefore, multimedia educational resources on LDKT may be a way forward to address the educational needs identified by candidates and recipients, donors, caregivers, and HCPs in the current review [80]. 

Several multimedia resources are currently available for kidney patients on the pathway to transplantation. The *KidneyTime* educational animations, developed by Kayler and colleagues in the US [47], comprises 12 animated videos about the LDKT process, benefits, and risks. These videos were developed in collaboration with kidney transplant candidates and recipients, donors, patient support networks, HCPs, experts, and stakeholders. Feedback suggests that the animations are suitable, acceptable, and usable to diverse groups of candidates and recipients, donors, and support networks [47, 63]. However, feedback from UK and USA versions of The *KidneyTime*, identified the lack of live action video content, suggesting video education may be improved with a combination of animation and live action footage or testimonials [47, 81, 82]. Rosaasen and colleagues in Canada sought to incorporate a much wider range of sources to ensure enriched educational content including animations to convey complex medical information, clinic and patient footage to familiarize the clinical environment, and testimonials from kidney patients, caregivers, donors, and HCPs to convey a storytelling approach [25]. Positive evidence from their RCT suggests adopting a similar format would be beneficial in the context of LDKT [26] and should form the basis of future patient educational resources in kidney transplantation. Regarding culturally diverse populations, a culturally sensitive video-based intervention by Arriola and colleagues in the USA reported no significant differences in LDKT knowledge compared to control among Black/African American patients [83]. The authors postulate that the lack of success was due to the flexible approach of the intervention, with participants given the option of which ‘tabs’ on the web-based intervention they wanted to access. As a result, participants will have gained differing levels of education, subsequently impacting the amount of knowledge they could gain.

Although the current review has several strengths, including adherence to Cochrane Rapid Review guidance [29, 30], incorporating a wide range of perspectives on LDKT, and use of thematic mapping to generate recommendations for LDKT education, there are some limitations. This review was conducted ‘rapidly’ to inform the development of LDKT educational videos, and as such, the date range was constrained to 2013–2023 and only three databases were searched. The results were limited to English language publications and high-income countries. Therefore, these results may not be generalizable to lower-middle-income countries. Further, ‘living’ was included as a search term, and it is possible some studies were missed if they did not include this in their key words or mesh terms. However, earlier qualitative reviews have been conducted on similar topics, which may provide further evidence on educational needs identified in pre-2013 studies [66, 84]. Another limitation is the absence of a comprehensive and systematic quality appraisal, which restricts our ability to provide a thorough evaluation of the methodological rigor and potential biases. Future research might work towards identifying which specific HCPs (e.g., physician, living donor coordinator, transplant coordinator, etc.) are best placed to deliver LDKT education, or whether a team-based approach in collaboration with prior transplant recipients and donors is best. Further, it would be pertinent to interpret quantitative findings in relation to LDKT education in order to establish effectiveness of specific education components.

### Conclusion

In the treatment of end-stage kidney disease, LDKT is considered the ‘gold standard’, particularly in terms of life expectancy and quality of life [68]. However, some transplant candidates may lose access to this gold standard treatment not because they do not have a viable donor, but because they have not received sufficient LDKT education. To overcome this barrier, LDKT education should address the needs identified in this review: be comprehensive and clear and delivered early in the kidney disease pathway, incorporate diverse, inclusive, and culturally sensitive materials, address communication barriers, include further information on postoperative support and health, provide group education sessions and access to peer support, and recognize the importance of family and friends. Multimedia educational resources, such as videos, testimonials, and animation, can provide easy-access supplements to hospital-based education for patients from all backgrounds.

### Practice implications

Addressing the LDKT educational needs of kidney transplant candidates and living donors is of critical importance to achieve the best outcomes for patients with chronic kidney disease. This review provides a synthesis of salient LDKT educational needs identified by kidney transplant candidates and recipients and their support networks, living donors, and HCPs. Providers involved in educating candidates and donors on LDKT may wish to incorporate findings from the current review to ensure they are delivering data-driven, high-quality education that addresses the needs of these patients. Researchers and HCPs must consider the barriers associated with the successful delivery of LDKT education to patients from diverse cultural and geographical backgrounds, and the best platform for delivering such content. Use of innovative educational formats to suit all learning capabilities, such as multimedia resources, is also encouraged.

## Data Availability

Data sharing is not applicable to this article as no datasets were generated or analysed during the current study.

## Abbreviations

APOL1: Apolipoprotein L1
CKD: Chronic Kidney Disease
COREQ: Consolidated Criteria for Reporting Qualitative Health Research
HCP’s: Healthcare professionals
LDKT: Living donor kidney transplantation
PRISMA: Preferred Reporting Items for Systematic Reviews and Meta-Analyse
RCT: Randomized Controlled Trial
